# Effect of Cytochrome P450 3A Inhibition and Induction by Itraconazole and Rifampin on Tazemetostat Pharmacokinetics in Patients With Advanced Malignancies

**DOI:** 10.1002/cpdd.1543

**Published:** 2025-05-10

**Authors:** Yingxue Chen, Renli Teng, Attila Szanto, Apoorva Kapopara, Rajat Bannerji, Julien Ogier, Devalingam Mahalingam

**Affiliations:** ^1^ IPSEN BIOSCIENCE INC Cambridge MA USA; ^2^ CareCeutics, LLC Berwyn PA USA; ^3^ Ipsen Innovation Les Ulis France; ^4^ Robert H. Lurie Comprehensive Cancer Center Chicago IL USA

**Keywords:** CYP3A induction, CYP3A inhibition, drug‐drug interaction, pharmacokinetics, tazemetostat

## Abstract

This study (NCT04537715) investigated itraconazole (strong cytochrome P450 [CYP] 3A inhibitor) and rifampin (strong CYP3A inducer) on tazemetostat pharmacokinetics. In Part 1, patients received tazemetostat 400 mg orally on Days 1, 15, and 36, and 400 mg twice daily on Days 3‐14 and Days 21‐35. Itraconazole 200 mg orally once daily was administered on Days 18‐38. In Part 2, patients received tazemetostat 800 mg orally once daily on Days 1, 15, and 24, and 800 mg twice daily on Days 3‐14 and Days 17‐23. Rifampin 600 mg orally once daily was administered on Days 17‐25. Twenty‐one patients in each part completed had plasma concentrations quantified for pharmacokinetic assessments. Itraconazole coadministration resulted in higher tazemetostat exposures after single doses (Day 21/Day 1) and steady state (Day 36/Day 15). Compared with tazemetostat alone, itraconazole increased mean maximum plasma concentration (C_max_) and area under the concentration‐time curve from time 0 to 12 hours (AUC_0‐12h_) by 2.00‐ and 3.12‐fold, respectively, after single doses. Following twice‐daily dosing, itraconazole increased mean steady‐state C_max_ and AUC_0‐12h_ by 1.86‐ and 2.47‐fold, respectively. Rifampin coadministration decreased tazemetostat steady‐state (C_max_) and AUC_0‐12h_ by approximately 84% (Day 24/Day 15). Itraconazole increased tazemetostat exposure by 2‐3‐fold, and rifampin decreased tazemetostat exposure by 84%, indicating that coadministration of tazemetostat with strong CYP3A inhibitors or inducers should be avoided.

Epigenetic regulation of gene expression via DNA methylation, histone modification, and chromatin remodeling is a dynamic and reversible process that does not involve alterations to underlying DNA sequences.^1^, ^2^ Aberrant histone modifications have been observed in many cancer types as they lead to inappropriate activation of oncogenes and inactivation of tumor suppressors, contributing to tumor initiation and progression.^3^, ^4^, ^5^ Given the role of epigenetic aberrations in tumorigenesis and reversibility of alterations, investigation and development of epigenetic inhibitors has attracted extensive attention.^6^, ^7^

Enhancer of zeste homolog 2 (EZH2) is a histone methyltransferase that has been identified as a catalytic subunit of polycomb repressive complex 2 for trimethylation of histone 3 at lysine 27, which is associated with repression of gene expression and involvement in tissue development and determination of stem cell fate.^1^, ^8^ EZH2 is aberrantly expressed in a variety of malignant tumors; therefore, EZH2 is an appropriate therapeutic target.^8^, ^9^

Tazemetostat is an oral methyltransferase inhibitor of EZH2 and the first‐in‐class epigenetic therapy approved by the US Food and Drug Administration for patients with follicular lymphoma and epithelioid sarcoma.^10^ Tazemetostat received accelerated approval based on the clinical activity demonstrated in 2 open‐label Phase II trials investigating tazemetostat in patients with histologically confirmed follicular lymphoma after 2 or more prior systemic therapies, and investigating tazemetostat in patients aged 16 years or older with histologically confirmed metastatic or locally advanced epithelioid sarcoma.^10^, ^11^, ^12^

Tazemetostat is absorbed rapidly once administered, with a median time to maximum plasma concentration (t_max_) of 1‐2 hours. The systemic exposure of tazemetostat is approximately dose proportional over the doses of 200‐1600 mg twice daily. In vitro, tazemetostat is mainly metabolized by cytochrome P450 (CYP), family 3, subfamily A (CYP3A) to form inactive metabolites, EPZ‐6930 and EPZ006931. EPZ‐6930 undergoes further metabolism by CYP3A.^3^, ^10^, ^13^ So that physicians can appropriately manage their patients, it is important to understand any potential drug‐drug interactions (DDIs) between tazemetostat and CYP3A inducers and inhibitors.

Rifampin is an antibiotic used for the treatment of pulmonary tuberculosis and an inducer of CYP isoforms; it is, particularly, a potent inducer of CYP3A. As a result, rifampin has been used extensively in clinical studies as a prototypical inducer of drug‐metabolizing enzymes.^14^ A typical DDI study utilizes a dose of rifampin 600 mg once daily, given orally.^15^, ^16^ Itraconazole is an antifungal used for the treatment of fungal infections and a strong inhibitor of CYP3A.^17^ Previous DDI studies have used doses of itraconazole ranging from 200 to 400 mg orally once daily. However, there is little difference in CYP3A inhibition between itraconazole 400 and 200 mg.^18^

Here, we present results from a DDI study (EZH‐108; NCT04537715), which evaluated the effect of CYP3A inhibition by itraconazole and CYP3A induction by rifampin on the pharmacokinetic (PK) and safety profile of tazemetostat in patients with advanced treatment‐refractory malignancies.

## Methods

### Study Design

This was a Phase I, multicenter, open‐label, 2‐part study encompassing a fixed‐sequence design that aimed to characterize the PK of tazemetostat when administered as single and twice‐daily oral doses alone or in combination with either itraconazole (Part 1) or rifampin (Part 2). Secondary objectives were to evaluate the safety profile of tazemetostat when coadministered with either itraconazole (Part 1) or rifampin (Part 2).

The study was performed at 10 investigational sites across United States (n = 7) and Spain (n = 3). The investigation sites in the United States were California Cancer Associates for Research and Excellence, Inc. (Encinitas, CA), the Angeles Clinical and Research Institute (Los Angeles, CA), Northwestern University‐Robert H. Lurie Comprehensive Cancer Center (Chicago, IL), South Texas Accelerated Research Therapeutics Midwest (Grand Rapids, MI), Gabrail Cancer Center (Canton, OH), University of Cincinnati Medical Center (Cincinnati, OH), and Mary Crowley Cancer Research (Dallas, TX). The institutional review boards (IRBs) of the sites in the United States were WIRB/Copernicus Group (WCG) IRB (Puyallup, WA), Mary Crowley Medical Research Center Institutional Review Board (Dallas, TX), and Salus IRB (Austin, TX). The investigational sites in Spain were Hospital Universitario Vall d'Hebron (Barcelona), Hospital Fundacion Jimenez Diaz (Madrid), and Onkologikoa (Donostia). The independent ethics committee (IEC) for the sites in Spain was CEIm La Fe Valencia (Valencia).

The study protocol and amendments and informed consent form and updates were reviewed and approved by an IEC or IRB before commencement of the study and during the study where applicable. The study was conducted under the provisions of the Declaration of Helsinki Version 2013, in accordance with the International Conference on Harmonization Consolidated Guideline on Good Clinical Practice, Food and Drug Administration Title 21 Part 312 and in compliance with IECs/IRBs and informed consent regulations. All patients enrolled in the study provided written, informed consent.

### Study Population

Eligible patients were adults aged 18 years or older with histologically and/or cytologically confirmed advanced metastatic or unresectable solid tumors that had progressed after treatment and for which there were no standard therapies available, or with histologically and/or cytologically confirmed hematologic malignancies, who had relapsed or were refractory, following 2 or more standard lines of systemic therapy and for which there were no standard therapies available. All eligible patients had an Eastern Cooperative Oncology Group performance status of 0‐2, had a life expectancy of greater than 3 months, had completed prior treatment before study entry, and were clinically stable at the time of consent.

Exclusion criteria included, but were not limited to, central nervous system or leptomeningeal metastasis, clinically significant bleeding diathesis, or coagulopathy, including known platelet function disorders, uncontrolled concurrent illness, or receiving medications that were known CYP3A inducers or inhibitors.

### Dosing Schedule and Administration

**Figure 1:**
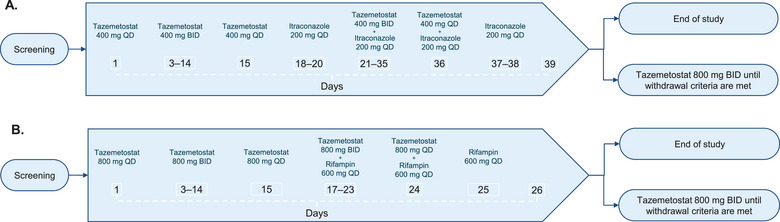
Study design for (A) Part 1: tazemetostat coadministered with itraconazole and (B) Part 2: tazemetostat coadministered with rifampin. BID, twice daily; QD, once daily.

Part 1 of the study evaluated the DDI between tazemetostat and itraconazole. On the mornings of Days 1 and 15, patients received a single dose of tazemetostat 400 mg orally. On Days 3‐14, patients received tazemetostat 400 mg twice daily (morning and evening). On Days 18‐20, patients received itraconazole 200 mg orally once daily in the morning. On Days 21‐35, tazemetostat 400 mg twice daily was coadministered in the morning with a single dose of itraconazole 200 mg. On Day 36, patients received a single dose of tazemetostat 400 mg coadministered in the morning with a single dose of itraconazole 200 mg. On Days 37 and 38, patients received a single dose of itraconazole 200 mg (Figure 1A). Throughout Part 1, administration of itraconazole occurred 1 hour after a meal.

Part 2 of the study evaluated the DDI between tazemetostat and rifampin. On the mornings of Days 1 and 15, patients received a single dose of tazemetostat 800 mg orally. On Days 3‐14, patients received tazemetostat 800 mg twice daily. On Days 17‐23, patients received tazemetostat 800 mg twice daily, coadministered in the morning with a single dose of rifampin 600 mg orally. On the morning of Day 24, patients received a single dose of tazemetostat 800 mg coadministered with rifampin 600 mg. On the morning of Day 25, patients received rifampin 600 mg (Figure 1B). Throughout Part 2, administration of rifampin occurred 1 hour before a meal.

In both Parts 1 and 2, patients who completed the study could either discontinue the study or continue oral tazemetostat treatment at the recommended therapeutic dose of 800 mg twice daily in 28‐day cycles from Day 40 until clinical progression, unacceptable toxicity, or until another discontinuation criterion was met.

### Pharmacokinetic Assessment

In Part 1, plasma concentrations of tazemetostat following tazemetostat dosing on Days 1, 15, 21, and 36 were quantified for PK analyses before dosing (0 hour), and at 0.5, 1, 1.5, 2, 4, 6, 8, 12, 24 (Days 21‐22), 36, 48 (Days 1‐3), and up to 72 hours after dosing (Days 15‐18 and Days 36‐39).

In Part 2, plasma concentrations of tazemetostat following tazemetostat dosing on Days 1, 15, and 24 were quantified for PK analyses before dosing (0 hour) and at 0.5, 1, 1.5, 2, 4, 6, 8, 12, 24, 36, and 48 hours.

Plasma concentrations of tazemetostat, over the ranges of 1.00‐2000 ng/mL, were quantified by a fully validated liquid chromatography with tandem mass spectrometric detection analytical method. Samples that had no detectable peak or had the calculated concentration <1.00 ng/mL were reported as below the lower limit of quantification. Tazemetostat analysis consisted of protein precipitation extraction and separation using mobile phases of 100 mM ammonium formate/water (1:9 v/v) (mobile phase A) and 100 mM ammonium formate/acetonitrile (1:9 v/v) (mobile phase B). Detection was achieved using an API‐5500 mass spectrometer (AB Sciex) using electrospray ionization (positive‐ion mode) in the multiple reaction monitoring mode. The multiple reaction monitoring mode m/z transitions monitored were 287.2/351.2 for tazemetostat and the dwell time was set at 300 milliseconds. The intra‐ and interassay accuracies and precisions for the tested concentrations were all within the defined acceptance criteria (±15.0% of nominal concentration at the lower limit of quantification and the coefficient of variation [CV] was 15.0% or less).

Plasma concentration‐time data for tazemetostat were analyzed by noncompartmental analysis using the validated software program Phoenix WinNonlin Professional Version 8.3 (Certara Inc., Mountain View, CA).

PK measures for tazemetostat included t_max_; the C_max_ occurring at t_max_; the area under the concentration‐time curve (AUC) from the last time of dosing to last measurable concentration estimated using the linear‐up/log‐down method (AUC_last_); AUC over the dosing interval from 0 to 12 hours after dosing, estimated using the linear‐up/log‐down method (AUC_0‐12h_); AUC extrapolated to infinity (AUC_0‐inf_), calculated as the sum of AUC from the last time of dosing to last measurable concentration and C_last_/λ_z_, where C_last_ is the last measurable concentration and λ_z_ is the first‐order rate constant associated with the terminal (log‐linear) portion of the curve; apparent terminal elimination half‐life (t_1/2_); C_max_ accumulation ratio, calculated as the ratio of C_max_ at steady‐state divided by C_max_ during the initial dosing interval (RacC_max_); and AUC accumulation ratio, calculated as the ratio of AUC_0‐12h_ at steady‐state divided by AUC_0‐12h_ during the initial dosing interval (RacAUC).

### Safety Analysis

Safety was assessed by monitoring treatment‐emergent adverse events (TEAEs) and vital signs, electrocardiography, and clinical laboratory tests. TEAEs were defined as adverse events that started or worsened in severity on or after the date of the first dose of the study drug through 30 days after the last dose. Treatment‐related TEAEs were summarized by each study drug for both study parts. Adverse events were coded using the Medical Dictionary for Regulatory Activities Version 23.1 and graded per Common Terminology Criteria for Adverse Events Version 5.0.

## Results

### Patient Population

A total of 42 patients were enrolled, with 21 patients receiving tazemetostat coadministered with itraconazole (Part 1) and 21 patients receiving tazemetostat coadministered with rifampin (Part 2). Of the 21 patients in each part, 16 patients had sufficient PK concentration data to calculate steady‐state PK parameters in Part 1 (Days 15 and 36) and Part 2 (Days 15 and 24) and did not have any major protocol deviations deemed to greatly alter the steady‐state exposure to tazemetostat, itraconazole, or rifampin. In Part 1, 16 (76.2%) patients completed the study. Five patients did not provide sufficient PK concentration data to calculate steady‐state PK parameters due to missed PK sample collections. In Part 2, 16 (76.2%) patients completed the study. Five patients did not provide sufficient PK concentration data to calculate steady‐state PK parameters. One patient was not administered rifampin due to AEs, 1 patient participated in the study for only 3 days, 2 patients were not administered rifampin and missed PK sample collections, and 1 patient missed the study drug doses. Baseline characteristics for Parts 1 and 2 are presented in Table S1.

In Part 1 (Cycle 1), the median duration of tazemetostat exposure was 5.14 weeks (range, 2.1‐5.1 weeks). Fifteen (71.4%) patients received 90% or more of the planned tazemetostat doses. Fourteen patients continued to receive tazemetostat from Cycle 2 onward.

In Part 2 (Cycle 1), the median duration of tazemetostat exposure was 3.43 weeks (range, 0.1‐3.7 weeks). Eighteen (85.7%) patients received 90% or more of the planned doses of tazemetostat. Seventeen patients continued to receive tazemetostat from Cycle 2 onward.

### Effect of Itraconazole on Tazemetostat PK

#### Single Dose (Day 21 vs Day 1)

**Figure 2:**
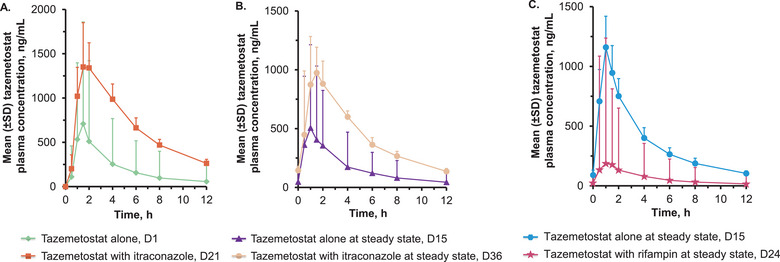
Tazemetostat plasma concentrations for Part 1 following coadministration of itraconazole with (A) single and (B) multiple doses of tazemetostat and for (C) Part 2 following coadministration of rifampin with multiple doses of tazemetostat. D, Day; SD, standard deviation.

Following single doses of tazemetostat with (Day 21) and without (Day 1) coadministration of itraconazole, tazemetostat exposure increased, with a least squares geometric mean (geoLSM) ratio of 2.00 and 3.12 for C_max_ and AUC_0‐12h_, respectively (Figure 2A). Tazemetostat t_1/2_ (geometric mean CV%) was shorter with (2.86 hours [28.9]) versus without (5.97 hours [25.6]) itraconazole coadministration (Table 1). Median tazemetostat t_max_ was similar with (1.53 hours) and without (1.44 hours) itraconazole coadministration.

**Table 1:** Summary of Tazemetostat PK, Coadministered With Either Itraconazole or Rifampin

	Part 1				Part 2		
PK assessment	Tazemetostat, single dose, Day 1	Tazemetostat, multiple dosing, Day 15	Tazemetostat, single dose with itraconazole, Day 21	Tazemetostat, multiple dosing with itraconazole, Day 36	Tazemetostat single dose, Day 1	Tazemetostat, multiple dosing, Day 15	Tazemetostat, multiple dosing with rifampin, Day 24
t_max_ (hour), median (range)	1.44 (0.50‐5.78)	1.27 (0.48‐5.83)	1.53 (0.92‐3.85)	1.49 (0.50‐4.00)	1.04 (0.97‐3.77)	1.00 (0.63‐4.00)	1.01 (0.00‐ 4.02)
C_max_ (ng/mL), mean (SD)	803 (433)	643 (443)	1640 (987)	1190 (647)	1550 (791)	1380 (990)	233 (239)
GeoMean (CV%)	704 (56.5)	543 (63.3)	1400 (67.3)	1010 (76.3)	1330 (67.4)	1100 (78.7)	181 (71.7)
AUC_last_ (ng•h/mL), mean (SD)	3250 (2730)	2340 (913)	7930 (4150)	6490 (2960)	6370 (3750)	5220 (3580)	934 (1280)
GeoMean (CV%)	2640 (68.9)	2200 (37.1)	6720 (73.4)	5750 (59.9)	5200 (89.5)	4270 (74.0)	676 (72.5)
AUC_0‐12_ _h_ (ng•h/mL), mean (SD)	2460 (1280)	1960 (800)	8000 (4150)	5160 (2370)	5310 (2890)	4330 (2740)	770 (955)
GeoMean (CV%)	2180 (55.3)	1830 (37.6)	6790 (73.2)	4530 (64.5)	4420 (83.2)	3610 (70.3)	588 (65.2)
t_1/2_ (hour), mean (SD)	6.13 (1.40)	8.62 (3.85)	2.95 (0.73)^a^	12.90 (5.19)	6.49 (1.55)	7.70 (1.58)	5.06 (1.39)
GeoMean (CV%)	5.97 (25.6)	8.01 (39.3)	2.86 (28.9)^a^	11.9 (43.8)	6.22 (35.6)	7.55 (20.3)	4.86 (31.7)
AUC_0‐12h_ ratio, geoLSM (90% CI)	NA		3.12 (2.44‐3.99)^b^	2.47 (2.02‐3.02)^c^	NA		0.163 (0.128‐0.208)^d^
C_max_ ratio, geoLSM (90% CI)	NA		2.00 (1.60‐2.48)^b^	1.86 (1.49‐2.31)^c^	NA		0.164 (0.122‐0.219)^d^
RacAUC, geoLSM (90% CI)	NA	0.842 (0.726‐0.976)^e^	NA			0.816 (0.638‐1.04)^e^	NA
RacC_max_, geoLSM (90% CI)	NA	0.771 (0.662‐0.897)^e^	NA			0.828 (0.635‐1.08)^e^	NA

AUC_last_, area under the concentration‐time curve from the last time of dosing to last measurable concentration; AUC_0‐12h_, area under the concentration‐time curve over the interval from 0 to 12 hours after dosing; C_max_, maximum plasma concentration; CV, coefficient of variation; geoLSM, least squares geometric mean; GeoMean, geometric mean; NA, not applicable; PK, pharmacokinetic; RacAUC, observed accumulation ratio; RacC_max_, C_max_ accumulation ratio; SD, standard deviation; t_max_, time to maximum plasma concentration; t_1/2_, apparent terminal elimination half‐life.

a Estimated over a 12‐hour dosing interval.

b Day 21 versus Day 1.

c Day 36 versus Day 15.

d Day 24 versus Day 15.

e Day 15 versus Day 1.

#### Steady State (Day 36 vs Day 15)

Itraconazole coadministration with tazemetostat resulted in higher tazemetostat exposure at steady state (Day 36) than tazemetostat alone (Day 15) (Figure 2B). The geoLSM ratios for C_max_ and AUC_0‐12h_ were 1.86 and 2.47, respectively. Tazemetostat t_1/2_ (geometric mean [CV%]) was slightly longer with (11.9 hours [43.8]) versus without (8.01 hours [39.3]) itraconazole coadministration (Table 1). Median tazemetostat t_max_ was slightly later with (1.49 hours) versus without (1.27 hours) itraconazole coadministration.

Following multiple doses of tazemetostat twice daily alone, the mean tazemetostat exposure (Day 15) was lower than those after a single tazemetostat dose (Day 1), with geoLSM ratios of 0.771 and 0.842 for RacC_max_ and RacAUC_0‐12h_, respectively (Table 1).

### Effect of Rifampin on Steady‐State PK of Tazemetostat (Day 24 vs Day 15)

Rifampin coadministration with tazemetostat (Day 24) resulted in lower tazemetostat exposure at steady state than tazemetostat administered alone (Day 15) (Figure 2C). The geoLSM ratios for C_max_ and AUC_0‐12h_ were 0.164 and 0.163, respectively. Tazemetostat t_1/2_ (geometric mean [CV%]) was shorter with (4.86 hours [31.7]) versus without (7.55 hours [20.3]) rifampin coadministration (Table 1). Median tazemetostat t_max_ was similar with (1.01 hours) and without (1.00 hour) rifampin coadministration.

Following multiple doses of tazemetostat twice daily alone, the mean tazemetostat exposure (Day 15) was lower than after a single tazemetostat dose (Day 1), with geoLSM ratios of 0.828 and 0.816 for RacC_max_ and RacAUC_0‐12h_, respectively (Table 1).

### Safety

In Part 1, 20 (95.2%) patients reported at least 1 TEAE, of which 13 (61.9%) patients experienced a TEAE considered related to the study drug by an investigator (Table S2). The most frequently reported TEAEs (occurring in 20% or more of subjects) in Part 1 were decreased appetite and dyspnea (33% each), anemia, fatigue, and nausea (28.6% each), and constipation (23.8%). Treatment‐related Grade 3/4 TEAEs experienced in Part 1 were anemia (n = 2; 9.5%; related to tazemetostat or itraconazole in both patients), and hyponatremia (n = 1; 4.8%; related to tazemetostat). Two deaths were reported in patients due to serious TEAEs (lung adenocarcinoma and superior vena cava syndrome) within 30 days following the last dose of the study drug in patients receiving tazemetostat with itraconazole; neither of these was considered treatment related.

In Part 2, all patients reported at least 1 TEAE, of which 18 (85.7%) experienced a TEAE considered related to the study drug by an investigator (Table S2). The most frequently reported TEAEs were fatigue (42.9%), diarrhea (33.3%), cough (28.6%), anemia, nausea, and constipation (23.8% each). Tazemetostat‐related Grade 3/4 TEAEs experienced in Part 2 were diarrhea (n = 1; 4.8%), alanine aminotransferase increased (n = 1; 4.8%), aspartate aminotransferase (n = 1; 4.8%), syncope (n = 1; 4.8%), acute kidney injury (n = 1; 4.8%), hypertension, and anemia (n = 2; 9.5%). Decreased lymphocyte count (n = 2; 9.5%) was a Grade 3/4 TEAE related to tazemetostat in 1 patient and rifampin in the other patient. There were no deaths resulting from TEAEs in Part 2.

## Discussion

This Phase I, 2‐part study was conducted to characterize the PK of oral tazemetostat when administered as a single and twice‐daily dose alone or in combination with itraconazole (Part 1) or rifampin (Part 2) in patients with advanced malignancies.

In Part 1, coadministration of the strong CYP3A inhibitor itraconazole resulted in higher exposures of tazemetostat after a single dose (Day 21 vs Day 1) and at steady state (Day 36 vs Day 15). When administered as a single dose, itraconazole increased the mean C_max_ and AUC_0‐12h_ by 2.00‐ and 3.12‐fold, respectively, compared with tazemetostat alone. At steady state, coadministration increased the mean C_max_ by 1.86‐fold and AUC_0‐12h_ by 2.47‐fold. Median t_max_ was similar in the presence or absence of itraconazole with single‐dose tazemetostat or steady state, whereas t_1/2_ was approximately 4 hours longer at steady state with coadministration of itraconazole. This increase in tazemetostat exposure is expected with coadministration of a strong CYP3A inhibitor, given that tazemetostat is mainly metabolized by CYP3A in vitro.^10^ However, the magnitude of tazemetostat exposure increases in the presence of itraconazole was significantly lower than expected from a strong inhibitor. It is recommended that the coadministration of tazemetostat with a strong CYP3A inhibitor should be avoided, and when coadministration is unavoidable, the tazemetostat dose should be reduced.^19^ However, despite this recommendation against coadministration with strong CYP3A inhibitors, it is important to acknowledge their varying inhibitory potencies. Tazemetostat coadministration with drugs with greater CYP3A inhibitory activity, such as ketoconazole, ritonavir, or cobicistat,^20^ may have resulted in a more substantial increase in tazemetostat exposure.

In Part 2, coadministration of the strong CYP3A inducer rifampin resulted in lower exposure to tazemetostat at steady state (Day 24 vs Day 15). Tazemetostat steady‐state exposure decreased by approximately 84% when coadministered with steady‐state rifampin. The geoLSM ratios for C_max_ and AUC_0‐12h_ were 0.164 and 0.163, respectively. This suggests that tazemetostat is very sensitive to rifampin induction of CYP3A, justifying the avoidance of concomitant administration with strong CYP3A inducers.^21^

Across both Parts 1 and 2, the nature and severity of TEAEs experienced were consistent with the known safety profile of tazemetostat. There were no protocol‐defined adverse events of special interest, and no new potential safety signals were identified during the study. The limited median duration of tazemetostat exposure, with most patients discontinuing the study due to disease progression, is consistent with a heavily pretreated patient population with advanced malignancies.

A limitation of this study was that sample sizes for each part of the study were relatively small given the large variability of tazemetostat PK assessments.

Overall, the results of this study reinforce that caution should be taken with concomitant administration of tazemetostat with strong CYP3A inhibitors or inducers due to their effect on tazemetostat exposure.

## Conflicts of Interest

Yingxue Chen, Attila Szanto, Apoorva Kapopara, Rajat Bannerji, and Julien Ogier are employees of, and may own stock/stock options in, Ipsen. Renli Teng is a partner of CareCeutics LLC, contracted by Ipsen. Devalingam Mahalingam has received research funding from Amgen, Merck, Oncolytics, and Rafael; has participated in a scientific advisory board for Actuate, Qurient; has participated in an advisory/speaker bureau for Amgen, BMS, Eisai, and Exelixis; has received funding paid to their institution from Acepodia, Actuate Therapeutics, ADC Therapeutics, Amgen, AVEO, Bayer, Blueprint Medicines, BMS, BioNTech, Dialectic Therapeutics, Epizyme, Fujifilm, ImmuneSensor, Immune‐Onc Therapeutics, Leap Therapeutics, Lycera Corp, Merck, Millennium, MiNA Alpha, NGM Biopharmaceuticals, Novartis, Oncolytics, Orano Med, Puma, Qurient, Rafael, Repare Therapeutics, Triumvira Immunologics, Vigeo Therapeutics, and Werewolf Therapeutics.

## Funding

This study was sponsored by Epizyme, an Ipsen company.

Where applicable, data from eligible studies are available 6 months after the studied medicine and indication have been approved in the United States and European Union or after the primary manuscript describing the results has been accepted for publication; whichever is later. Further details on Ipsen's sharing criteria, eligible studies, and process for sharing are available here: https://vivli.org/members/ourmembers/. Any requests should be submitted to www.vivli.org for assessment by an independent scientific review board.

## Supporting information



Supporting Information

Supporting Information

## Data Availability

Qualified researchers may request access to patient‐level study data that underlie the results reported in this publication. Additional relevant study documents, including the clinical study report, study protocol with any amendments, annotated case report form, statistical analysis plan, and data set specifications may also be made available. Patient‐level data will be anonymized, and study documents will be redacted to protect the privacy of study participants.
