# Supplementary material for: Effect of Cytochrome P450 3A Inhibition and Induction by Itraconazole and Rifampin on Tazemetostat Pharmacokinetics in Patients With Advanced Malignancies
Source: Clin Pharmacol Drug Dev. 2025 May 10;14(7):520–7. doi: 10.1002/cpdd.1543 (PMC12209990; doi:10.1002/cpdd.1543)
Supplement: Supplementary file 1 — Supporting Information [file CPDD-14-520-s001.pdf]

Supplemental Digital Content for: Effect of CYP3A inhibition and induction by itraconazole and rifampin on tazemetostat pharmacokinetics in patients with advanced malignancies

Supplementary Table S1. Baseline characteristics

**Baseline characteristics**	**Part 1 (*N* = 21)**	**Part 2 (*N* = 21)**
Median age, years (range)	62.0 (21–82)	64.0 (35–78)
Sex, n (%)		
Female	8 (38.1)	13 (61.9)
Male	13 (61.9)	8 (38.1)
Race, n (%)		
White	18 (85.7)	12 (57.1)
Asian	2 (9.5)	3 (14.3)
Black or African American	0	2 (9.5)
Not reported/Other	1 (4.8)	4 (19.0)
ECOG performance status, n (%)		
0	11 (52.4)	14 (66.7)
1	10 (47.6)	7 (33.3)
Tumor type, n (%)		
Advanced solid tumor	20 (95.2)	21 (100.0)
Adenocarcinoma of unknown primary	1 (4.8)	0
Adrenal cortical carcinoma	0	1 (4.8)
Basal cell carcinoma	1 (4.8)	0
Bladder cancer	0	1 (4.8)
Breast cancer	1 (4.8)	0
Cholangiocarcinoma	1 (4.8)	2 (9.5)
Colorectal cancer	4 (19.0)	2 (9.5)
Endometrial cancer	1 (4.8)	1 (4.8)
Ewing sarcoma	1 (4.8)	0
Head and neck cancer	3 (14.3)	0
Malignant melanoma of choroid of right eye	0	1 (4.8)
Melanoma	0	1 (4.8)
Mesothelioma lung cancer	1 (4.8)	2 (9.5)
Mucinous adenocarcinoma of bilateral lung	1 (4.8)	
Neuroendocrine tumors	0	1 (4.8)
NSCLC – Adenocarcinoma of the right lung with bone and soft tissue metastases	1 (4.8)	0
NSCLC	1 (4.8)	1 (4.8)
Ovarian cancer	0	2 (9.5)
Pancreatic cancer	1 (4.8)	1 (4.8)
Prostate cancer	0	1 (4.8)
Small cell lung cancer carcinoma	1 (4.8)	0
Thyroid carcinoma	1 (4.8)	0
Thymoma	0	1 (4.8)
Urachal adenocarcinoma	0	1 (4.8)
Uterine carcinosarcoma	0	1 (4.8)
Uterine leiomyosarcoma	0	1 (4.8)
Hematopoietic neoplasm	1 (4.8)	0
DLBCL	1 (4.8)	0
Stage at diagnosis, n (%)		

I	0	1 (4.8)
II	3 (14.3)	0
III	1 (4.8)	3 (14.3)
IV	12 (57.1)	13 (61.9)
Unknown	4 (19.0)	4 (19.0)
Other	1 (4.8)	0
Median time since last disease progression, months	0.87	1.22
Median number of prior lines of systemic anticancer therapy	4	3

DLBCL, diffuse large B-cell lymphoma; ECOG, Eastern Cooperative Oncology Group;

NSCLC, non-small cell lung cancer.

Supplementary Table S2. Summary of TEAEs in patients treated with tazemetostat

**Patients, n (%)**	**Part 1 (*N* = 21)**	**Part 2 (*N* = 21)**
Any TEAE	20 (95.2)	21 (100.0)
Any TEAE Grade 3 or 4	11 (52.4)	13 (61.9)
Any treatment-related TEAEs	13 (61.9)	18 (85.7)
Tazemetostat	12 (57.1)	16 (76.2)
Itraconazole	8 (38.1)	N/A
Rifampin	N/A	8 (38.1)
Any treatment-related TEAE Grade 3 or 4	2 (9.5)	7 (33.3)
Tazemetostat	2 (9.5)	7 (33.3)
Itraconazole	1 (4.8)	NA
Rifampin	NA	1 (4.8)
Any TEAE leading to dose reduction	0	2 (9.5)
Any TEAE leading to study drug interruption	9 (42.9)	7 (33.3)
Any TEAE leading to study drug discontinuation	1 (4.8)	0
Any TESAE	8 (38.1)	9 (42.9)
Any treatment-related TESAE	0	1 (4.8)
Tazemetostat	0	1 (4.8)
Itraconazole	0	NA
Rifampin	NA	0

TEAEs were defined as adverse events that started or worsened in severity on or after the date of first dose of study drug through 30 days after the last dose. Adverse events were coded using Medical Dictionary for Regulatory Activities Version 23.1 and graded per Common Terminology Criteria for Adverse Events Version 5.0.

NA, not applicable to the study part; TEAE, treatment-emergent adverse event; TESAE, treatment-emergent serious adverse event.
