# Supplementary material for: Effect of Cytochrome P450 3A Inhibition and Induction by Itraconazole and Rifampin on Tazemetostat Pharmacokinetics in Patients With Advanced Malignancies
Source: Clin Pharmacol Drug Dev. 2025 May 10;14(7):520–7. doi: 10.1002/cpdd.1543 (PMC12209990; doi:10.1002/cpdd.1543)
Supplement: Supplementary file 2 — Supporting Information [file CPDD-14-520-s002.pdf]

Plain language summary for Chen Y, et al. Effect of CYP3A inhibition and induction by itraconazole and rifampin on tazemetostat pharmacokinetics in patients with advanced malignancies

Tazemetostat is a medicine approved by the US Food and Drug Administration (FDA) for patients with specific cancers: follicular lymphoma (a type of white blood cell cancer) or epithelioid sarcoma (a rare cancer usually found in the soft tissues of the body, such as the arms or legs). Once tazemetostat is absorbed and used by the body, it is broken down in the liver by an enzyme (a protein that helps to speed up chemical reactions) to make it easier for the body to get rid of it. The enzyme that breaks down tazemetostat is called CYP3A. Other medicines can slow down or speed up how quickly CYP3A works. A medicine that slows down how quickly CYP3A works is called a CYP3A inhibitor and a medicine that speeds up how quickly CYP3A works is called a CYP3A inducer. An example of a CYP3A inhibitor is itraconazole, and an example of a CYP3A inducer is called rifampin. The aim of this study was to find out how much itraconazole and rifampin affect the absorption and processing of tazemetostat in adults with advanced cancer. To do this, scientists measured the amount of tazemetostat in the blood when taken either with or without itraconazole (Part 1) or rifampin (Part 2).

In Part 1, 21 patients were given tazemetostat and itraconazole at the same time. Itraconazole given at the same time as tazemetostat increased the level of tazemetostat in the body by two to three times that seen with tazemetostat given alone. The increase in tazemetostat level caused by itraconazole in the blood could cause more side effects to happen to patients.

In Part 2, 21 patients were given tazemetostat and rifampin at the same time. Rifampin given at the same time as tazemetostat decreased the level of tazemetostat in the body by roughly 84% of that seen with tazemetostat given alone. The decrease in

tazemetostat level caused by rifampin in the body could stop tazemetostat from working properly against cancer.

Healthcare providers should be careful and monitor patients when giving patients tazemetostat at the same time as strong CYP3A inhibitors like itraconazole or strong CYP3A inducers like rifampin.

Studies like this help researchers understand what medicines healthcare providers can give at the same time with other medicines, and what medicines, when given together, should be carefully monitored or avoided.
